# A normative modeling approach to quantify white matter changes and predict functional outcomes in stroke patients

**DOI:** 10.3389/fnins.2024.1334508

**Published:** 2024-02-05

**Authors:** Houming Su, Su Yan, Hongquan Zhu, Yufei Liu, Guiling Zhang, Xiaolong Peng, Shun Zhang, Yuanhao Li, Wenzhen Zhu

**Affiliations:** Department of Radiology, Tongji Hospital, Tongji Medical College, Huazhong University of Science and Technology, Wuhan, China

**Keywords:** white matter microstructure, normative modeling, Mahalanobis distance, prognosis, stroke

## Abstract

**Objectives:**

The diverse nature of stroke necessitates individualized assessment, presenting challenges to case-control neuroimaging studies. The normative model, measuring deviations from a normal distribution, provides a solution. We aim to evaluate stroke-induced white matter microstructural abnormalities at group and individual levels and identify potential prognostic biomarkers.

**Methods:**

Forty-six basal ganglia stroke patients and 46 healthy controls were recruited. Diffusion-weighted imaging and clinical assessment were performed within 7 days after stroke. We used automated fiber quantification to characterize intergroup alterations of segmental diffusion properties along 20 fiber tracts. Then each patient was compared to normative reference (46 healthy participants) by Mahalanobis distance tractometry for 7 significant fiber tracts. Mahalanobis distance-based deviation loads (MaDDLs) and fused MaDDL_multi_ were extracted to quantify individual deviations. We also conducted correlation and logistic regression analyses to explore relationships between MaDDL metrics and functional outcomes.

**Results:**

Disrupted microstructural integrity was observed across the left corticospinal tract, bilateral inferior fronto-occipital fasciculus, left inferior longitudinal fasciculus, bilateral thalamic radiation, and right uncinate fasciculus. The correlation coefficients between MaDDL metrics and initial functional impairment ranged from 0.364 to 0.618 (*p* < 0.05), with the highest being MaDDL_multi_. Furthermore, MaDDL_multi_ demonstrated a significant enhancement in predictive efficacy compared to MaDDL (integrated discrimination improvement [IDI] = 9.62%, *p* = 0.005) and FA (IDI = 34.04%, *p* < 0.001) of the left corticospinal tract.

**Conclusion:**

MaDDL_multi_ allows for assessing behavioral disorders and predicting prognosis, offering significant implications for personalized clinical decision-making and stroke recovery. Importantly, our method demonstrates prospects for widespread application in heterogeneous neurological diseases.

## Introduction

Stroke is one of the leading causes of death and disability in the world, resulting in an enormous burden on society and the economy (Feigin et al., 2014). Cerebral white matter (WM) is particularly susceptible to damage in stroke. Disruption of WM integrity hinders the transmission and communication of nerve signals, resulting in impairment of diverse neurological functions, and encompassing motor and sensory deficits as well as cognitive impairments (Wang et al., 2016). Furthermore, the integrity and connectivity of WM serve as indicators for the condition of axonal networks and can provide insights into stroke prognosis (Lindenberg et al., 2012).

Diffusion magnetic resonance imaging is used to characterize cerebral WM microstructure (Chen et al., 2020). Region of interest (ROI) analysis has revealed MW microstructural differences associated with motor deficits (Ingo et al., 2020). Tract-based spatial statistics (TBSS) analyses and probabilistic fiber tracking analyses are commonly employed methods to quantify white matter integrity and connectivity patterns. TBSS analysis has identified abnormalities in the corpus callosum and bilateral corticospinal tracts (CST), whereas probabilistic fiber tracking analyses revealed abnormalities in the left thalamic radiation, inferior fronto-occipital fasciculus, and bilateral CST (Li et al., 2015). However, these group-level findings only represent a subset of patients and may not account for crucial individual differences. Given the multifocal and heterogeneous nature of stroke, new techniques are required for individualized assessment of abnormal deviations along fiber tracts.

The normative model represents an emerging approach for evaluating intersubject imaging differences by quantifying deviations from a normal distribution. A notable application of this model is Mahalanobis distance tractometry (MaD-Tract), a novel framework for evaluating individual-level differences in fiber tracts (Guerrero-Gonzalez et al., 2022). Mahalanobis distance (MaD), a method quantifying the distance between a point and a distribution, is characterized by the number of standard deviations that a point is away from the mean of a distribution. It incorporates covariance between variables in multi-dimensional measures and maintains scale invariance, as expressed by the following equation:


$$\mathrm{MaD}=\sqrt{{\left(X_{i}-\mu \right)}^{T}\sum^{-1}\left(X_{i}-\mu \right)}$$

Here, $X_{i}$ represents the set of multivariate neuroimaging metrics for each subject, μ corresponds to the mean of the multivariate distribution of neuroimaging metrics, and Σ is the variance-covariance matrix among metrics. MaD has proven informative in diverse neuroimaging applications, including signal outlier detection (Guerrero-Gonzalez et al., 2022), identification of brain variation in neurological diseases (Dean et al., 2017), and assessment of WM maturational processes (Li et al., 2022). Moreover, multivariate analysis derived from MaD-Tract demonstrates greater reliability compared to conventional univariate analysis (Dean et al., 2017; Owen et al., 2021). Hence, MaD may offer advantages in detecting individual brain differences in heterogeneous disease, such as stroke. Automated fiber quantification (AFQ) can automatically identify and locate fiber tracts (Yeatman et al., 2012; Chen et al., 2020). However, the integration of AFQ and MaD analyses for stroke patients at both group and individual levels remains unexplored.

In this study, we first identified seven damaged fiber tracts in the stroke group through intergroup comparison using the AFQ technique on 3D T1BRAVO and diffusion MRI. We then focused on assessing outlier deviation along these injured fiber tracts using MaD analysis. Furthermore, we performed correlation analyses for dysfunction and prognostic regression analyses. Our objective was to evaluate stroke-induced white matter microstructural abnormalities at individual levels and identify potential prognostic biomarkers.

## Methods

### Participants

This prospective study was conducted according to the guidelines of the Declaration of Helsinki and approved by the institutional review boards at our hospital. All participants signed written informed consent before study participation. Forty-six unilateral stroke patients were recruited consecutively from 2020 to 2022. The diagnosis of stroke was based on the clinical manifestations such as limb numbness, limb weakness, dizziness, and discomfort, along with typical MRI appearance during the acute phase. Inclusion criteria for patients were: (1) the first-onset ischemic stroke (within 7 days after symptom onset), (2) infarction restricted to the left basal ganglia and/or surrounding areas, (3) right-handedness before stroke. Exclusion criteria for patients were: (1) MRI contraindications; (2) tumor, intracranial hemorrhage, or other neurological disorders. Forty-six age-and sex-matched subjects without a history of neurological or psychiatric disorders were included as HC participants.

All patients underwent the National Institutes of Health Stroke Scale (NIHSS) evaluation and received standard MRI imaging on the same day during the acute period. At 3 months, they were evaluated by the modified Rankin Scale (mRS) and were dichotomized into good (mRS score ≤ 2) and poor (mRS score > 2) categories.

### MRI imaging

All images were acquired using a 3.0 T MRI scanner (Discovery MR 750, GE Medical Systems, Waukesha, WI) equipped with a 32-channel head coil. Conventional MR sequences including DWI (spin echo-echo planar sequence, repetition time/echo time = 3,000/65.5, matrix = 256 × 256, slice thickness = 5 mm, field of view (FOV) = 24 cm, *b* value = 1,000 s/mm^2^) and 3D T1BRAVO (repetition time/echo time = 7.1/2.7 ms, matrix = 256 × 256, slice thickness = 1.0 mm, FOV = 256 × 256 mm^2^) were obtained. Diffusion MRI was acquired with *b*-value of 0, 1,250, and 2,500 s/mm^2^ (25 noncollinear diffusion directions for each nonzero *b*-value, repetition time/echo time = 6,500/85 ms, matrix = 128 × 128, slice thickness = 3 mm, spacing = 0 mm, FOV = 256 mm × 256 mm).

### Ischemic lesion overlaps analysis

**Figure 1:**
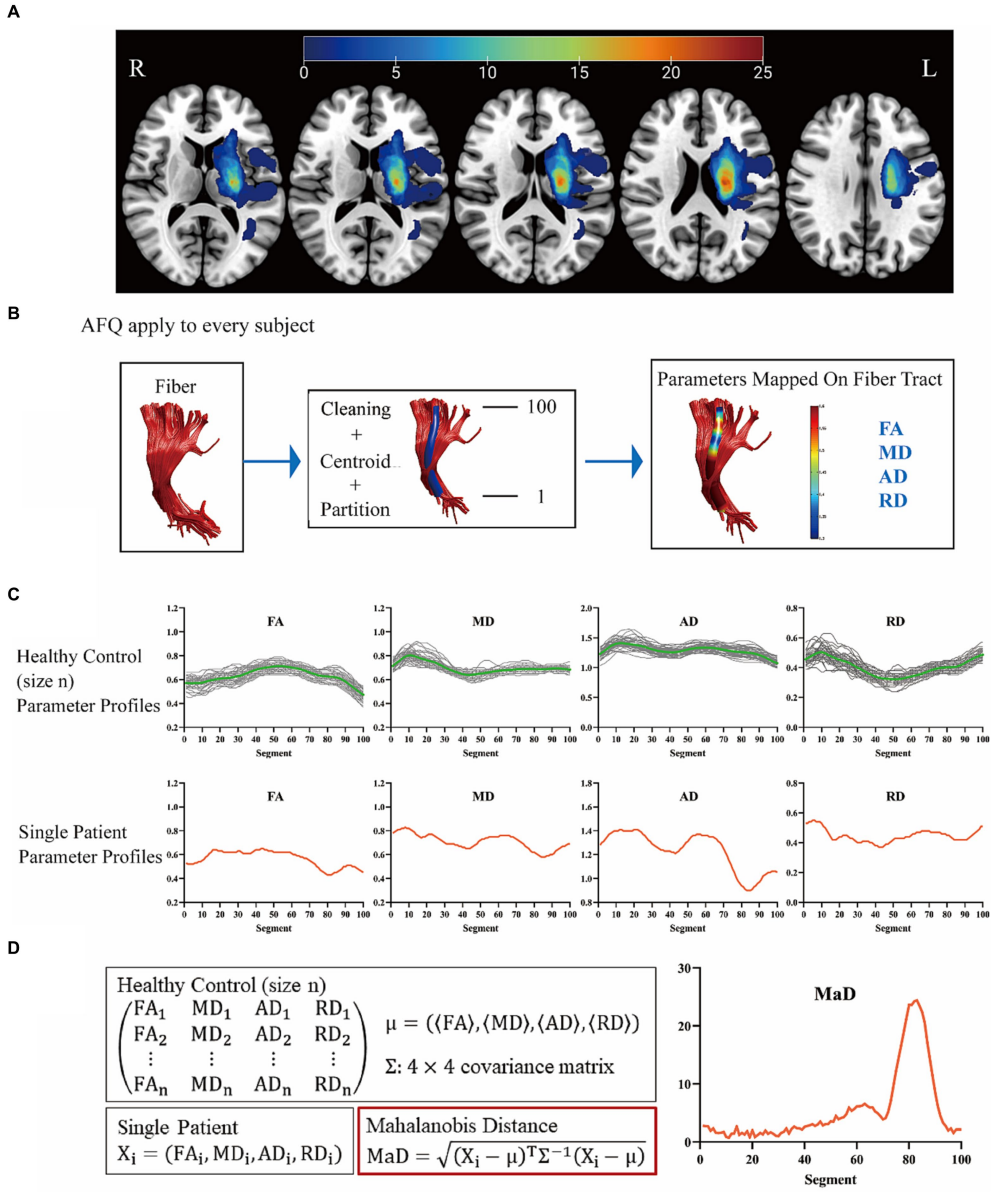
The workflow of this study. **(A)** Stroke lesion map. Unilateral hemispheric stroke lesions overlapped in 46 patients. The colored bars indicated the number of patients with lesions in the voxels. **(B)** Twenty main white matter fiber tracts were analyzed. **(C,D)** As an example, the corticospinal tract has been shown here. L, left side; R, right side; AFQ, automated fiber quantification; FA, fractional anisotropy; MD, mean diffusivity; AD, axial diffusivity; RD, radial diffusivity; MaD, Mahalanobis distance.

To delineate lesion regions on conventional DWI images, a neuroradiologist (H.S., 3.5 years of experience) meticulously labeled the lesions using ITK-SNAP.^1^ Subsequently, the lesion masks and T1-weighted images were spatially normalized using SPM12.^2^ Finally, all lesion masks in the MNI space were overlapped to generate the stroke lesion map (Figure 1A).

### Automated fiber quantification procedure

The flow chart is shown in Figure 1B. The diffusion images with higher *b*-values and diffusion gradients in multiple directions underwent preprocessing using the tractoflow pipeline. This pipeline encompassed the following steps: (1) denoising with Marchenko-Pastur principal component analysis; (2) correction for Gibbs ringing artifacts using Mrtrix3^3^; (3) motion and eddy current artifact correction, and skull stripping by FMRIB Software Library software (University of Oxford, Oxford, UK); (4) N4 correction on b = 0 mm^2^/s by ANTs (N4BiasFieldCorrection); (5) application of the bias field to the entire diffusion imaging data; (6) fitting of diffusion metrics by FSL DTIFIT with single shell (b = 1,250 s/mm^2^). The diffusion metrics were further quantified along the tract trajectory by the AFQ package in MATLAB R2012b (MathWorks, Inc., Natick, Massachusetts, United States) (Yeatman et al., 2012). Outliers in each subject were identified and removed using specific criteria, including deviations (> 5 standard deviations) from the tract core or lengths exceeding (> 4 standard deviations) the mean fiber length determined by the Gaussian distribution. Twenty major WM fiber tracts were analyzed, including bilateral corticospinal tract, bilateral thalamic radiation, bilateral cingulum hippocampus, bilateral cingulum cingulate, callosum forceps minor, callosum forceps major, bilateral inferior fronto-occipital fasciculus, bilateral inferior longitudinal fasciculus, bilateral superior longitudinal fasciculus, bilateral arcuate fasciculus, bilateral uncinate fasciculus. All these 20 fiber tracts were carefully checked per subject. One hundred equidistant nodes along each tract core were segmented, and the diffusion metrics (FA, MD, axial diffusivity [AD], radial diffusivity [RD]) were extracted.

### MaD-tract analysis

The MaD-Tract framework is shown in Figures 1C,D. Only the 7 fiber tracts with intergroup differences in FA, MD, AD, or RD were used for MaD-Tract analysis. The MaD of each segment can be computed for each patient as described by Guerrero-Gonzalez et al. (2022). The analyses were carried out at a significance level of 0.05/100 (Bonferroni correction, 100 segments per tract), yielding a crucial MaD >5.49. If the MaD value exceeded the threshold, the segment was considered abnormal. The area of the MaD curve above the threshold was depicted as Mahalanobis distance-based deviation load (MaDDL).

### Statistical analysis

The demographic and clinical information were analyzed using the one-way ANOVA, Mann–Whitney U test, or Chi-square test by the SPSS 22.0 software package (SPSS, Inc., Chicago, IL, United States). FA, MD, AD, and RD were compared between groups along each tract with statistical significance set at *p* < 0.05/20 after false discovery rate (FDR) correction. A heat map of MaD and a scatter plot of the MaDDL for each patient were plotted. The MaDDLs of the 7 fiber tracts were combined into a new indicator, MaDDL_multi_. Pearson correlation analyses were performed between the MaDDL of 7 fiber tracts, MaDDL_multi_, FA_CST_L_, and the NIHSS score. Furthermore, partial correlation analyses were conducted to control for the influence of sex and age. In Model 1, we used these 8 MaDDL metrics and FA_CST_L_ to perform univariate logistic regression to predict 3-months mRS. In Model 2, gender and age were included as covariates to obtain predictions for the linear regression of the 8 MaDDL metrics as well as FA_CST_L_. Subsequently, logistic regression analyses were performed on these predicted values to predict 3-months mRS separately. Finally, receiver operating characteristic (ROC) analyses were performed for both models. *p* < 0.05 after FDR correction was used in both the correlation analyses and logistic regression analyses. In addition, we computed the integrated discrimination improvement (IDI) to assess the incremental predictive value of MaDDL_multi_ compared to other MaDDL metrics and FA_CST_L_.

## Results

### Demographic data and clinical characteristics

As shown in Table 1, there were no significant differences in age (*p* = 0.522) or sex (*p* = 0.832). The median volume of the ischemic lesion was 3.36 cm^3^ (interquartile range, 1.71–7.76 cm^3^). The median (inter-quartile range) days from stroke onset to imaging was 3 (inter-quartile range: 2, 4). During the acute period, the median NIHSS score was 4 (interquartile range, 2–8), and at follow-up, 69.57% of patients achieved an mRS score < 3. Among these patients, 45.65% (*n* = 21) had hypertension, 21.74% (*n* = 10) had diabetes, 4.35% (*n* = 2) had coronary artery disease, and 2.17% (*n* = 1) had atrial fibrillation. Figure 1A presented the characteristics of the stroke lesion with infarction in the left basal ganglia and/or surrounding areas.

**Table 1:** Demographic and clinical characteristics.

	BGS (*n* = 46)	HC (*n* = 46)	*p* value
Age (years)	58.89 ± 10.62	58.54 ± 6.35	0.849
Sex (female)	19 (41.30%)	18 (39.13%)	0.832
Lesion volume (cm^3^)	3.36 (1.71, 7.76)	NA	
NIHSS	4 (2, 8)	NA	
3-months mRS (score < 3)	32 (69.57%)	NA	
Days between stroke onset and MRI	3 (2, 4)	NA	
Hypertension	21 (45.65%)	NA	
Diabetes	10 (21.74%)	NA	
Coronary artery disease	2 (4.35%)	NA	
Atrial fibrillation	1 (2.17%)	NA	

NIHSS, National Institute of Health Stroke Score; mRS, modified Rankin scale; MRI, magnetic resonance imaging; BGS, basal ganglia stroke; HC, health control. Values were presented as n (%), mean ± standard deviation, or median (inter-quartile range).

### Group differences in WM fiber tracts by AFQ

**Figure 2:**
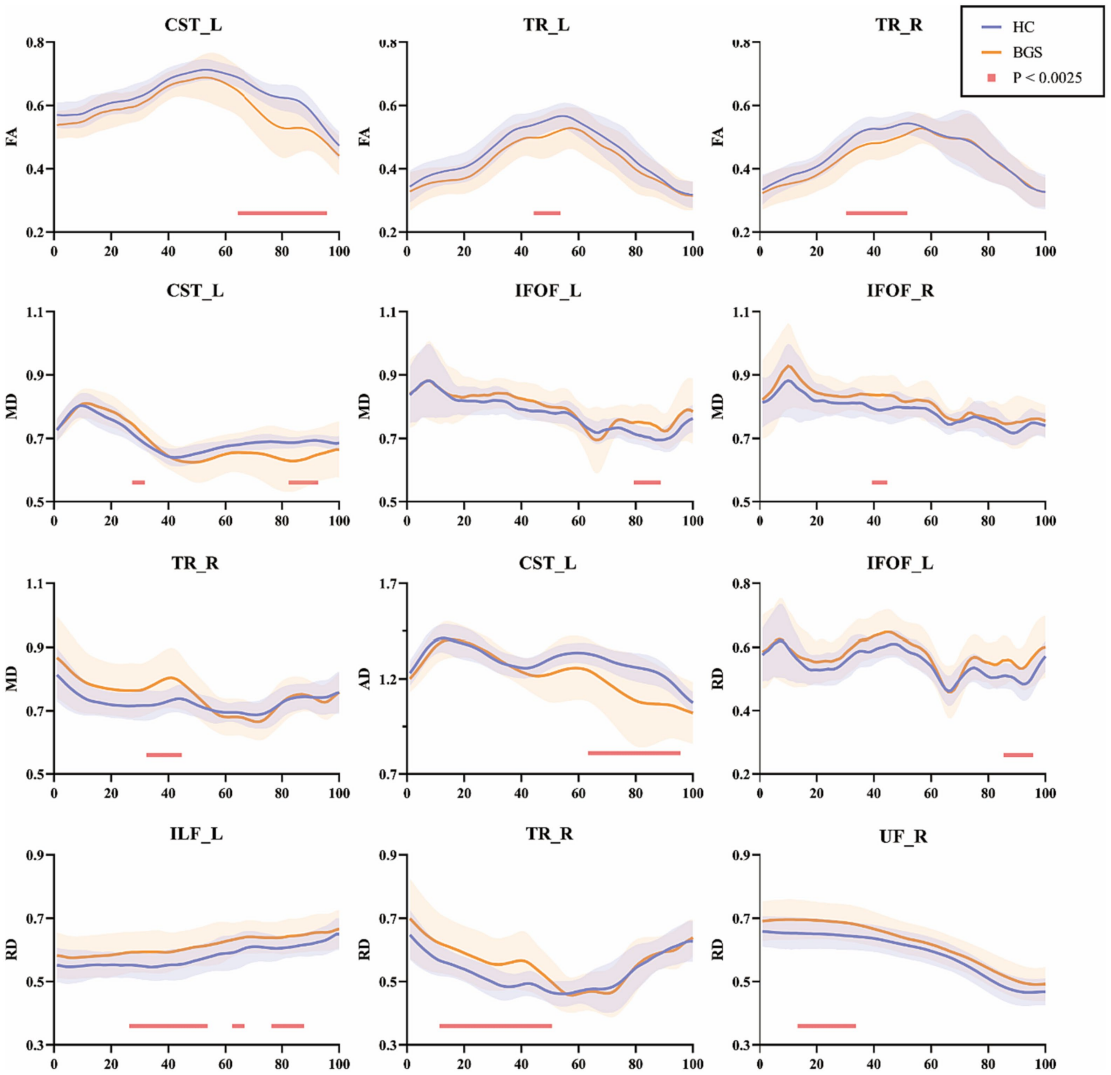
Pointwise comparison of diffusion parameters between groups of 20 white matter fiber tracts (only those with differences were shown). The blue lines represented the HC group and the orange lines represented the BGS group (solid lines for means and shading for standard deviations). The pink lines showed significantly changed segments (*p* < 0.05/20, FDR correction). HC, healthy controls; BGS, basal ganglia stroke; CST_L, left corticospinal; TR_L, left thalamic radiation; TR_R, right thalamic radiation; IFOF_L, left inferior fronto-occipital fasciculus; IFOF_R, right fronto-occipital fasciculus; ILF_L, left inferior longitudinal fasciculus; UF_R, right uncinate fasciculus.

Details of fiber tracts tracking were shown in supplementary information (Supplementary Table S1). FA of the BGS group showed a significant decrease when compared to HC (Figure 2), including (1) left corticospinal tract (CST_L, node 65–95); (2) left thalamic radiation (TR_L, node 45–53); (3) right thalamic radiation (TR_R, node 31–51; *p* < 0.05/20, FDR correction). There was no significant change in other fiber tracts.

MD of the BGS group showed significant alterations: (1) CST_L (node 28–31 and node 83–92); (2) left inferior fronto-occipital fasciculus (IFOF_L, node 80–88); (3) right inferior fronto-occipital fasciculus (IFOF_R, node 40–44); (4) TR_R (node 33–44; *p* < 0.05/20, FDR correction).

As for AD, the BGS group showed a significant reduction in CST_L (node 64–95; *p* < 0.05/20, FDR correction). In addition, the BGS group showed significantly decreased RD in IFOF_L (node 86–95), left inferior longitudinal fasciculus (ILF_L, node 27–53, node 65–66, and node 77–87), TR_R (node 12–50) and right uncinate fasciculus (UF_R, node 14–33; *p* < 0.05/20, FDR correction).

### Assessment of WM damage by MaD-Tract analysis

**Figure 3:**
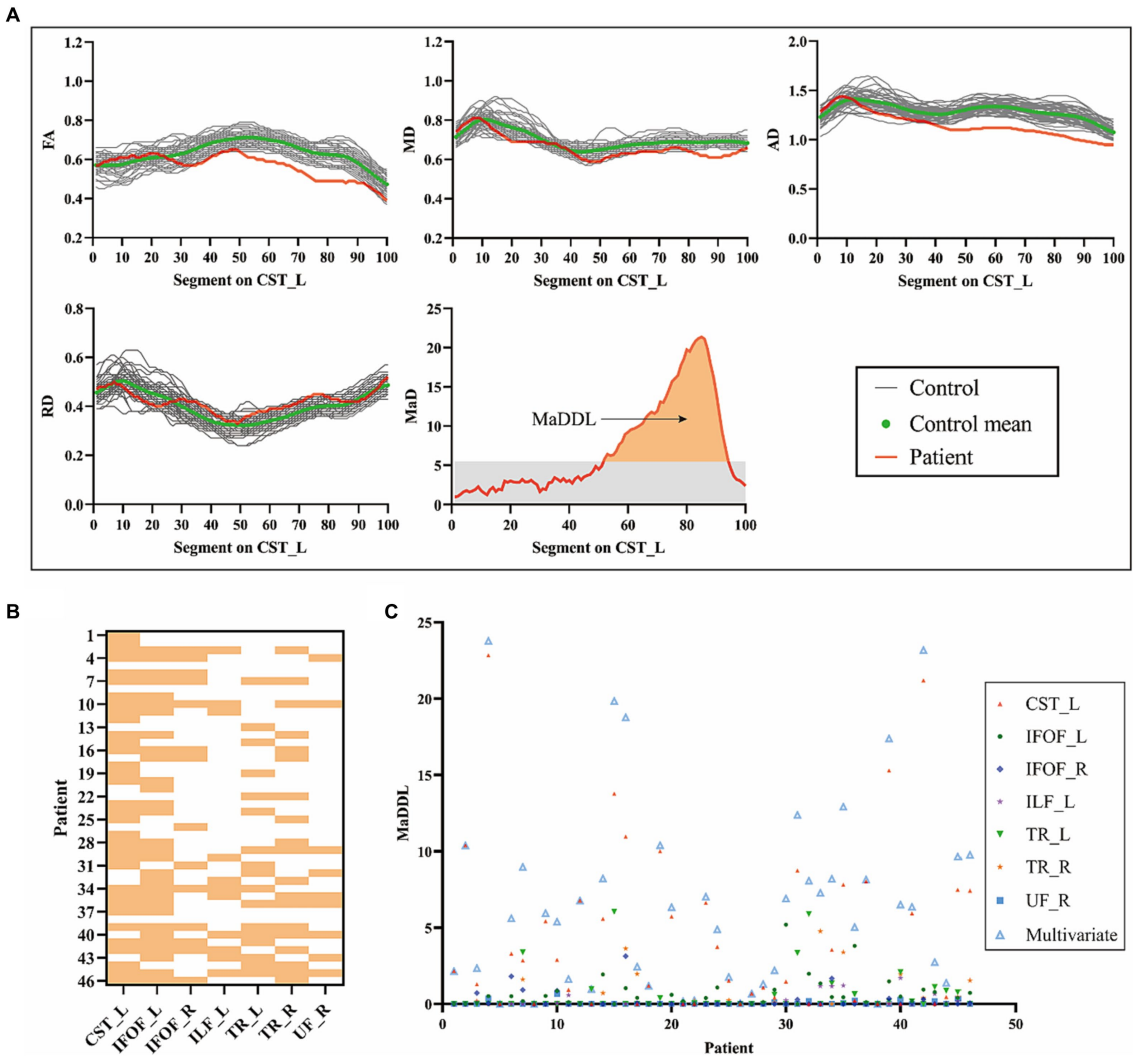
Detection of patient-specific microstructural abnormalities with MaD-Tract. **(A)** The example presented the 4 univariate diffusion parameter distributions of the left corticospinal tract for one patient and the healthy control group. Segments that exceeded the anomaly index threshold (gray shading in the MaD plot) were labeled as anomalous. The orange area of the MaD curve above the threshold was depicted as MaDDL. **(B)** Heat map of the distribution of structural abnormalities by MaD tract analysis in each patient across the seven abnormal fiber tracts. If at least one segment is marked as abnormal, it is highlighted. **(C)** Scatter plot of the MaDDL for each patient. MaD, Mahalanobis distance; MaDDL, Mahalanobis distance-based deviation load.

Figure 3A showed an example of the diffusion metrics of CST_L for one patient and the HC group. In this case, the patient had significantly lower FA, MD, and AD than the HC group. There were anomalous MaD values between node 52 and node 94, with a MaDDL of 5.58. As shown in Figure 3B, WM microstructural abnormalities occurred predominantly in CST_L (34/46), followed by IFOF_L (30/46). Figure 3C showed MaDDLs for each patient.

### Correlation analysis between MaDDL metrics and NIHSS

**Figure 4:**
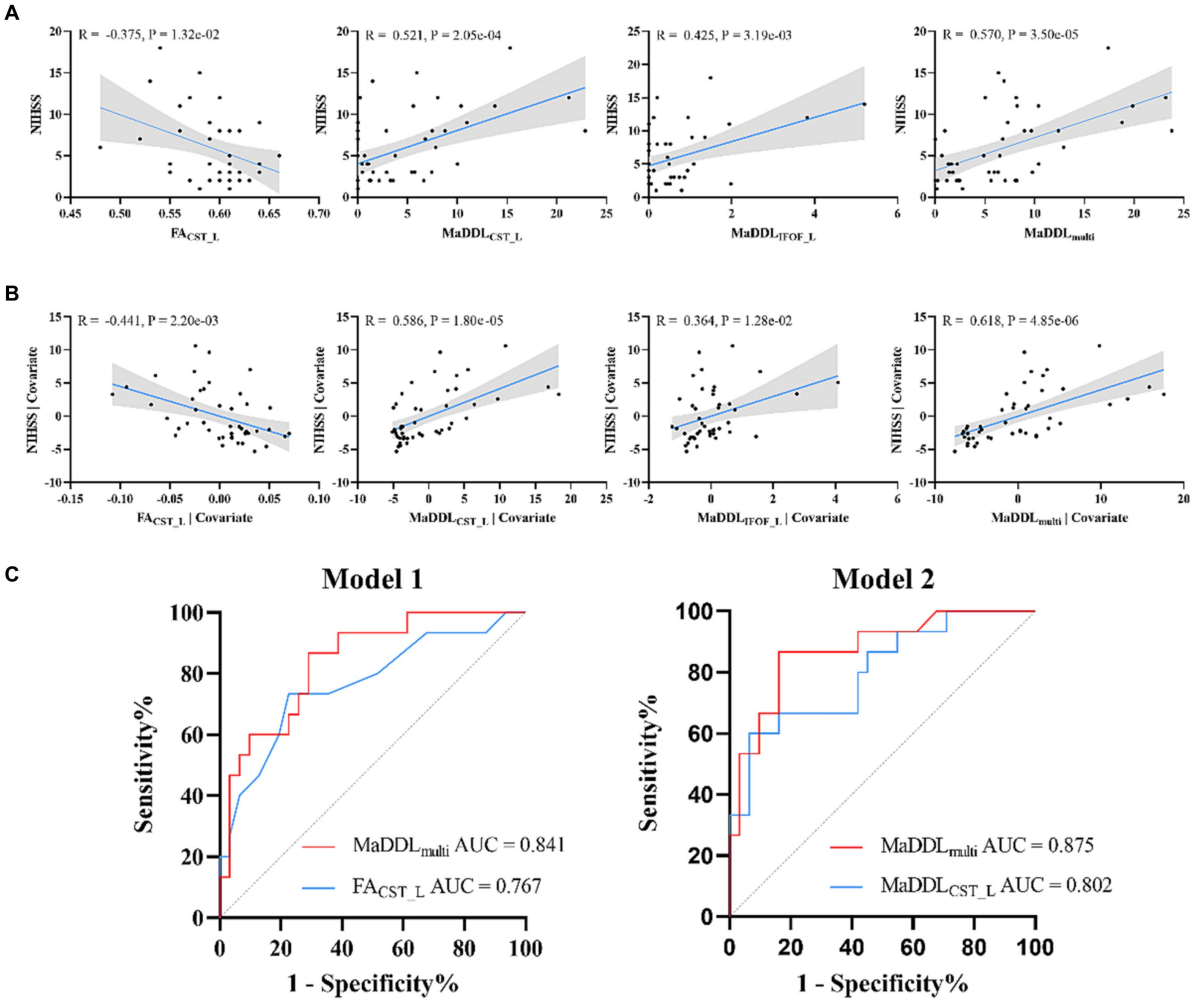
**(A)** Scatterplots and correlations for NIHSS and MaDDL metrics as well as FA_CST_L_ from Pearson correlation analyses. **(B)** Scatterplots and correlations for NIHSS and MaDDL metrics as well as FA_CST_L_ from partial correlation analyses with age and sex as covariates. **(C)** MaDDL_multi_ and FA_CST_L_ regressed univariately against 3-month mRS (Model 1). MaDDL_CST_L_ and MaDDL_multi_ regressed against 3-month mRS with age and sex as covariates (Model 2). In addition, MaDDL_multi_ demonstrated a significant enhancement in predictive efficacy compared to MaDDL_CST_L_ (IDI = 9.62%, *p* = 0.005) and FA_CST_L_ (IDI = 34.04%, *p* < 0.001).

As Pearson correlation analyses in Figure 4A showed, FA_CST_L_ (*R* = −0.375), MaDDL_IFOF_L_ (*R* = 0.425), MaDDL_CST_L_ (*R* = 0.521), and MaDDL_multi_ (*R* = 0.570) had correlations with the NIHSS scores (*p* < 0.05 after FDR correction). As for partial correlation analyses (Figure 4B), compared with FA_CST_L_, MaDDL_IFOF_L_, and MaDDL_CST_L_, MaDDL_multi_ achieved the larger correlation coefficient (*R* = 0.618, *p* < 0.05 after FDR correction). No significant correlation between MaDDL and NIHSS scores was observed for the other fiber tracts.

### Logistic regression analysis for predicting mRS at 3 months

In the univariate logistic regressions (Model 1 in Table 2 and Figure 4C), FA_CST_L_ and MaDDL_multi_ showed significantly predictive value (*p* < 0.05 after FDR correction). In the logistic regressions with age and sex as covariates (Model 2), MaDDL_CST_L_ and MaDDL_multi_ were significantly associated with mRS (*p* < 0.05 after FDR correction). The area under the ROC curve (AUC) for these valuable metrics discriminating poor from good outcomes ranged from 0.767 to 0.875, with sensitivities from 0.600 to 0.867 and specificities from 0.710 to 0.935. MaDDL_multi_ in Model 2 exhibited an inspiring predictive performance (AUC = 0.875, 95% confidence interval: 0.767–0.984), and stroked a balance between sensitivity (0.867) and specificity (0.839). Notably, MaDDL_multi_ demonstrated an improvement in predictive efficacy compared to MaDDL_CST_L_ (IDI = 9.62%, *p* = 0.005) and FA_CST_L_ (IDI = 34.04%, *p* < 0.001).

**Table 2:** Logistic regression analysis for predicting functional outcomes in 3 months.

Metric	Sensitivity	Specificity	AUC (95% CI)	*p* value
**Univariate logistic regression analysis (Model 1)**				
MaDDL_CST_L_	0.867	0.581	0.742 (0.581, 0.903)	0.008
MaDDL_IFOF_L_	0.800	0.581	0.674 (0.506,0.842)	0.058
MaDDL_IFOF_R_	0.600	0.871	0.714 (0.545, 0.883)	0.020
MaDDL_ILF_L_	0.400	0.839	0.628 (0.445, 0.811)	0.163
MaDDL_TR_L_	0.200	0.935	0.523 (0.341, 0.704)	0.806
MaDDL_TR_R_	0.533	0.903	0.731 (0.563, 0.900)	0.012
MaDDL_U_R_	0.200	0.968	0.561 (0.376, 0.746)	0.504
MaDDL_multi_	0.867	0.710	0.841 (0.724, 0.958)	<0.001*
FA_CST_L_	0.774	0.733	0.767 (0.615, 0.919)	0.004*
**Multivariate logistic regression analysis (Model 2)**				
MaDDL_CST_L_	0.600	0.935	0.802 (0.664, 0.940)	0.001*
MaDDL_IFOF_L_	0.667	0.774	0.753 (0.601,0.904)	0.006
MaDDL_IFOF_R_	0.533	0.839	0.709 (0.539, 0.878)	0.023
MaDDL_ILF_L_	0.533	0.839	0.710 (0.545, 0.875)	0.022
MaDDL_TR_L_	0.667	0.710	0.709 (0.546, 0.871)	0.023
MaDDL_TR_R_	0.533	0.968	0.751 (0.587, 0.914)	0.006
MaDDL_U_R_	0.733	0.645	0.720 (0.560, 0.881)	0.016
MaDDL_multi_	0.867	0.839	0.875 (0.767, 0.984)	<0.001*
FA_CST_L_	0.710	0.667	0.706 (0.542, 0.871)	0.024

AUC, area under the receiver operating characteristic curve; CI, confidence interval. *Statistical significance after correction for multiple testing (*p* < 0.05 after FDR correction).

## Discussion

In this study, we applied the normative model to quantitatively assess WM microstructural damage in stroke patients at the individual level. The results showed: (1) significant WM abnormalities found in specific segments of the CST_L, IFOF_L, IFOF_R, ILF_L, TR_L, TR_R, and UF_R; (2) the subject-level map of MaDDLs; (3) the correlation between the MaDDL metrics and NIHSS score; (4) the potential of MaDDL_multi_ as imaging markers for identifying favorable prognosis.

Microstructural changes in diffusion metrics varied along fiber tracts, with certain segments displaying heightened vulnerability. This vulnerability was believed to be influenced by diverse factors, such as fiber type, axonal diameter, packing density, and membrane permeability (Burzynska et al., 2010; Jones et al., 2013). Observed changes in FA, MD, AD, and RD might result from Wallerian WM degeneration, cytotoxic, and vasogenic edema (Haque et al., 2019; van Veluw et al., 2019; Sinke et al., 2021). Traditionally, studies focused on alterations of the mean measure within specific fiber tracts. For instance, the FA of entire corticospinal tracts significantly decreased in stroke patients, correlating with impaired function and prognostic recovery (Vargas et al., 2013; Wen et al., 2016; Lee et al., 2021). AFQ was applied to identify altered diffusion features in specific vulnerable segments along fiber tract (Zhang et al., 2018; Du et al., 2022). In this study, the affected tracts detected at the group level via AFQ belonged to two systems: the association WM fibers (IFOF_L, IFOF_R, ILF_L, UF_R) and the projection WM fibers (CST_L, TR_L, and TR_R). These damaged fiber tracts aligned with findings from previous studies (Yang et al., 2017; Yao et al., 2020; Kancheva et al., 2022). In this study, abnormal deviations were quantified by integrating four diffusion metrics in specific damaged segments of the fiber tracts. We found that MaDDL_multi_ outperformed FA_CST_L_, extracted by the conventional method, not only in the partial correlation coefficient with NIHSS (*R* = 0.618 vs. *R* = −0.441) but also in its predictive efficacy (IDI = 34.04%, *p* < 0.001). This implied that concentrating on the abnormal deviations in diffusion multivariate within damaged segments was more sensitive than the crude observation of changes in diffusion univariate across the entire fiber tract. One study demonstrated superior discriminatory power between traumatic brain injury and healthy controls using multivariate MaD analysis, compared to traditional univariate FA of either fiber tract (Taylor et al., 2020). Another study similarly highlighted the superiority of multivariate MaD analysis over traditional univariate diffusion measures in distinguishing individuals with or without autism spectrum disorder (Dean et al., 2017). Overall, multivariate MaD analysis can provide additional clinical insights compared to traditional univariate FA.

Brain disorders, such as stroke, are often complex, with diverse clinical and imaging heterogeneity (Bonkhoff et al., 2022). Tailored efficacy can be optimized when treatments align with each individual’s unique circumstances (Ingemanson et al., 2019). Yet, prevailing analysis predominantly focus on group-level averages, disregarding interindividual differences as inconsequential noise (Kapur et al., 2012). Neglecting this heterogeneity risks obscuring valuable biomarkers crucial for guiding personalized treatment strategies and prognostic evaluations. Normative modeling, an emerging approach, offers promise in addressing this challenge by mapping individual-level deviations from expected pattern (Marquand et al., 2016). This method holds the potential to enable meticulous scrutiny and profound comprehension of the intricate array of individual variations, shifting research emphasis from mean effects to exploring individual variations (Rutherford et al., 2022). We constructed a heat map illustrating abnormal deviations in damaged fiber tracts and a scatter plot indicating deviation loads for each patient. Notably, three patients (Patient 5, Patient 8, and Patient 38) exhibited a MaDDL of zero, all of whom exhibited a good prognosis. Future studies are expected to forecast favorable or adverse prognoses by establishing individual-level MaDDL thresholds using extensive sample data as a reference, thereby augmenting personalized clinical decision-making and stroke recovery. Our work represents a pioneering effort in applying normative modeling to stroke.

Notably, multivariate analysis is capable of identifying subtle alterations and improving prognosis prediction. As a multi-parameter fusion metric, MaD took advantage of the existing correlation of tensor diffusivities through covariance matrix estimation. This might provide additional information (Taylor et al., 2020; Guerrero-Gonzalez et al., 2022). Specifically, MaDDL_multi_ took into account not only CST_L damage but also other fiber tracts damage, which may produce incremental prediction value. For instance, MaDDL_multi_ demonstrated increased sensitivity in detecting WM changes compared to univariate MaDDL. Moreover, MaDDL_multi_ significantly improved the correlation coefficient in partial correlation analysis and exhibited enhanced predictive efficacy compared to other univariate MaDDL metrics. It meant that multivariate derived from normative modeling approaches might improve subject-level predictions.

This research has several potential limitations. Firstly, the sample size was relatively small, necessitating further validation through prospective studies involving larger populations. Secondly, due to the technical constraints of AFQ, only the central portion of traced fibers could be analyzed. Thus, the possibility that other parts may be important for clinical assessment and prognostic value cannot be excluded. Lastly, this study was cross-sectional in nature. Longitudinal studies should be needed to investigate changes in WM tracts with the recovery of neural function.

## Conclusion

We observed the vulnerability of microstructural integrity in specific segments of the CST_L, IFOF_L, IFOF_R, ILF_L, TR_L, TR_R, and UF_R. MaDDL_multi_ can comprehensively evaluate the impairment of all these injured fiber tracts, offering incrementally valuable insights. Thus, MaDDL_multi_ played a critical role in functional impairment and exhibited exceptional predictive capability for functional outcomes, surpassing the performance of MaDDL_CST_L_ alone or conventional FA_CST_L_. The predictive biomarkers have the potential to contribute to the functional recovery of stroke patients.

## Data availability statement

The original contributions presented in the study are included in the article/Supplementary material), further inquiries can be directed to the corresponding authors.

## Ethics statement

The studies involving humans were approved by Tongji Hospital, Tongji Medical College, Huazhong University of Science and Technology, Wuhan, China. The studies were conducted in accordance with the local legislation and institutional requirements. The participants provided their written informed consent to participate in this study.

## Author contributions

HS: Conceptualization, Formal analysis, Methodology, Writing – original draft, Writing – review & editing. SY: Investigation, Writing – review & editing. HZ: Data curation, Writing – review & editing. YLiu: Resources, Writing – review & editing. GZ: Visualization, Writing – review & editing. XP: Software, Writing – review & editing. SZ: Validation, Writing – review & editing. YLi: Conceptualization, Formal analysis, Methodology, Writing – review & editing. WZ: Funding acquisition, Project administration, Resources, Supervision, Writing – review & editing.

## Footnotes

^1^http://itksnap.org

^2^http://www.fil.ion.ucl.ac.uk/spm/software/spm12

^3^https://www.mrtrix.org
