# Supplementary material for: A normative modeling approach to quantify white matter changes and predict functional outcomes in stroke patients
Source: Front Neurosci. 2024 Feb 5;18:1334508. doi: 10.3389/fnins.2024.1334508 (PMC10877717; doi:10.3389/fnins.2024.1334508)
Supplement: Supplementary file 1 [file Table_1.docx]

**Supplementary Information**

**Table S1 |** Successful identification rate for each of 20 fiber tracts in HC and BGS

| Tract | Total subjects or raw samples (N0) | | No. of subjects showing successful tract identification (N1) | | Ratio (N1/N0) | |
| --- | --- | --- | --- | --- | --- | --- |
|  | HC | BGS | HC | BGS | HC (%) | BGS (%) |
| AF_L | 46 | 46 | 46 | 46 | 100 | 100 |
| AF_R | 46 | 46 | 45 | 45 | 97.83 | 97.83 |
| CC_L | 46 | 46 | 46 | 42 | 100 | 91.30 |
| CC_R | 46 | 46 | 46 | 42 | 100 | 91.30 |
| Forceps major | 46 | 46 | 46 | 42 | 100 | 91.30 |
| Forceps minor | 46 | 46 | 46 | 46 | 100 | 100 |
| CH_L | 46 | 46 | 43 | 40 | 93.48 | 86.96 |
| CH_R | 46 | 46 | 45 | 41 | 97.83 | 89.13 |
| CST_L | 46 | 46 | 46 | 46 | 100 | 100 |
| CST_R | 46 | 46 | 46 | 46 | 100 | 100 |
| IFOF_L | 46 | 46 | 46 | 46 | 100 | 100 |
| IFOF_R | 46 | 46 | 46 | 46 | 100 | 100 |
| ILF_L | 46 | 46 | 46 | 46 | 100 | 100 |
| ILF_R | 46 | 46 | 46 | 46 | 100 | 100 |
| SLF_L | 46 | 46 | 46 | 46 | 100 | 100 |
| SLF_R | 46 | 46 | 46 | 46 | 100 | 100 |
| TR_L | 46 | 46 | 46 | 46 | 100 | 100 |
| TR_R | 46 | 46 | 46 | 46 | 100 | 100 |
| UF_L | 46 | 46 | 46 | 46 | 100 | 100 |
| UF_R | 46 | 46 | 46 | 46 | 100 | 100 |

BGS, basal ganglia stroke; HC, health control; AF_L, left arcuate fasciculus; AF_R, right arcuate fasciculus; CST_L, left corticospinal tract; CST_R, right corticospinal tract; CC_L, left cingulum cingulate; CC_R, right cingulum cingulate; CH_L, left cingulum hippocampus; CH_R, right cingulum hippocampus; IFOF_L, left inferior fronto-occipital fasciculus; IFOF_R, right inferior fronto-occipital fasciculus; ILF_L, left inferior longitudinal fasciculus; ILF_R, right inferior longitudinal fasciculus; SLF_L, left superior longitudinal fasciculus; SLF_R, right superior longitudinal fasciculus; TR_L, left thalamic radiation; TR_R, right thalamic radiation; UF_L, left uncinate fasciculus; UF_R, right uncinate fasciculus.
